# A case of thyroid hormone resistance with thyroid regrowth: implications of misdiagnosis on patient care

**DOI:** 10.1210/jcemcr/luag034

**Published:** 2026-03-12

**Authors:** Matthew Shelly, Luke Miller, Katelyn Graver, Mohammad Ishaq Arastu

**Affiliations:** Lewis Katz School of Medicine, Temple University, Philadelphia, PA 19140, USA; Lewis Katz School of Medicine, Temple University, Philadelphia, PA 19140, USA; Lewis Katz School of Medicine, Temple University, Philadelphia, PA 19140, USA; Department of Endocrinology, St. Luke's University Health Network, Bethlehem, PA 18015, USA

**Keywords:** thyroid hormone resistance (RTH), thyroid hormone, recurrent goiter, misdiagnosis

## Abstract

We present the case of a male patient misdiagnosed with resistant hyperthyroidism who erroneously underwent total thyroidectomy complicated by thyroid tissue regrowth. Subsequent iodine-131 (I-131) radiation therapy and medical management further confounded the misdiagnosis. Serological examination in the following years revealed persistently elevated thyroid stimulating hormone (TSH) levels ranging from 45.7 μIU/mL (SI: 45.7 mIU/L) to 134.0 μIU/mL (SI: 134.0 mIU/L) (reference range, 0.550-5.00 μIU/mL [SI: 0.55-5.00 mIU/L]) with variable free thyroxine (FT4) levels ranging from 0.80 ng/dL to 8.1 ng/dL (SI: 10.3 pmol/L to 104.3 pmol/L) (reference range, 0.90-1.70 ng/dL [SI: 11.5-21.8 pmol/L]). Thyroid hormone resistance syndromes (RTH) are characterized by thyroid hormone resistance in organ tissues. Clinical presentation varies based on the severity of thyroid hormone dysregulation and the location of hormone resistance. RTH is often misdiagnosed due to variable phenotypic and biochemical presentations, resulting in erroneous medical and surgical treatments that further complicate patient management. The purpose of this report is to highlight the complex treatment course of this patient and describe challenges faced when diagnosing and managing RTH.

## Introduction

Resistance to thyroid hormone (RTH), historically known as Refetoff syndrome, is a predominantly inherited condition associated with decreased sensitivity to thyroid hormone (TH) occurring once in every 40 000 births [1]. RTH syndromes are subdivided into 2 clinical phenotypes, generalized RTH (GRTH) and pituitary RTH (PRTH), which correspond to pathogenic receptor variations in the thyroid hormone receptor beta 1 (*TRβ1*) and thyroid hormone receptor beta 2 (*TRβ2*) genes, respectively [2, 3]. PRTH is TH resistance confined to the pituitary gland, resulting in disturbances in the negative feedback loop of the hypothalamic-pituitary-thyroid axis. Biochemically, PRTH presents with elevated serum free thyroxine (FT4), free triiodothyronine (FT3), and a supranormal to elevated thyroid stimulating hormone (TSH) level [2]. Phenotypically, PRTH often resembles Graves disease with a prominent goiter and thyrotoxic symptoms, resulting in erroneous diagnosis and treatment in approximately one-third of patients [2, 4]. In GRTH, the TH resistance extends beyond the pituitary gland to include peripheral tissues. GRTH presents as hypothyroidism with elevated FT4 and TSH or euthyroid with supranormal FT4 and normal TSH based on the severity of TH resistance [2]. Accurate diagnosis of RTH is necessary to minimize inappropriate medical treatments and prevent unnecessary surgery. RTH should be included in the differential diagnosis for patients with a family history, discordant lab values, or treatment-resistant thyroid disease.

## Case presentation

In 1968, a 22-year-old man with no medical history presented to his primary care physician (PCP) and was noted to be underweight and thin. Family history was significant for 2 paternal aunts with thyroid goiter requiring thyroidectomy. The patient was found to be hyperthyroid based on elevated FT4 levels (levels unavailable). He was started on Lugol's iodine without improvement in symptomatology. He pursued a second opinion and underwent total thyroidectomy in 1970. Initially, the patient felt symptomatic improvement, but 4 months later developed hyperactivity with follow-up imaging revealing regrowth of thyroid tissue. In 1976, given continued symptoms, iodine-131 (I-131) radiation therapy with 6 millicuries was initiated with no symptomatic improvement. The following year, phenelzine 15 mg 3 times per day was prescribed by his PCP with notable improvement. In 1979, he was found to be hypothyroid and started on 75 mcg levothyroxine daily with no alteration in dosing until 2013. Due to the transition of his PCP out of practice, the patient sought specialized thyroid disease management with an endocrinologist.

## Diagnostic assessment

Initial physical exam by endocrinology in December 2011 was negative for thyroid goiter, heart palpitations, or gynecomastia. Laboratory values showed a FT4 of 1.0 ng/dL (SI: 12.9 pmol/L) (reference range, 0.90-1.70 ng/dL [SI: 11.5-21.8 pmol/L]), TSH of 48.37 μIU/mL (SI: 48.37 mIU/L) (reference range, 0.550-5.00 μIU/mL [SI: 0.55-5.00 mIU/L]), and FT3 of 2.7 pg/mL (SI: 3.5 pmol/L) (reference range, 2.3-4.0 pg/mL [SI: 3.53-6.14 pmol/L]) (Table 1). Anti-thyroglobulin and human anti-mouse antibodies were negative and thyroglobulin immunometric assay was 12.8 ng/mL (SI: 12.8 μg/L) (reference range 1.4-29.3 ng/mL [SI: 1.4-29.3 μg/L]). Due to elevated TSH, a pituitary magnetic resonance imaging (MRI) was obtained, which showed no suprasellar or pituitary mass. Repeat laboratory values in April 2023 showed similar values, including a FT4 of 1.1 ng/dL (SI: 14.2 pmol/L) and a TSH of 73.66 μIU/mL (SI: 73.66 mIU/L) (Table 1).

**Table 1:** Selected laboratory values throughout clinical course

Date	Levothyroxine dosage	TSH (0.550-5.00 µIU/mL) (0.55-5.00 mIU/L)	Free T3 (2.3-4.0 pg/mL) (3.53-6.14 pmol/L)	Free T4 (0.90-1.70 ng/dL) (11.5-21.8 pmol/L)	Thyroglobulin RIA (<40 ng/mL) (<40 µg/mL)	Thyroglobulin IMA (1.4-29.3 ng/mL) (1.4-29.3 µg/L)	Thyroglobulin antibody (0.0-0.9 IU/mL) (0.0-0.9 kIU/L)	Human anti-mouse Ab (0-138 ng/mL)	Thyroid stimulating immunoglobulin (0-139%)
Dec 16, 1978	NA	NA	NA	5.0 ng/dL (64.4 pmol/L)	NA	NA	NA	NA	NA
Mar 14, 1989	75 mcg daily	NA	NA	4.0 ng/dL (51.5 pmol/L)	NA	NA	NA	NA	NA
Mar 15, 1999	75 mcg daily	NA	NA	2.8 ng/dL (36.0 pmol/L)	NA	NA	NA	NA	NA
Jul 15, 2004	75 mcg daily	76.63 µIU/mL (76.63 mIU/L)	NA	8.1 ng/dL (104.3 pmol/L)	NA	NA	NA	NA	NA
Jul 1, 2005	75 mcg daily	50.1 µIU/mL (50.1 mIU/L)	NA	7.6 ng/dL (97.8 pmol/L)	NA	NA	NA	NA	NA
Nov 4, 2007	75 mcg daily	85 µIU/mL (85 mIU/L)	NA	1.0 ng/dL (12.9 pmol/L)	NA	NA	NA	NA	NA
Jul 26, 2010	75 mcg daily	134.0 µIU/mL (134.0 mIU/L)	NA	5.5 ng/dL (70.8 pmol/L)	NA	NA	NA	NA	NA
Dec 2, 2011	75 mcg daily	48.37 µIU/mL (48.38 mIU/L)	2.7 pg/mL (3.5 pmol/L)	1.0 ng/dL (12.9 pmol/L)	NA	12.8 ng/mL (12.8 µg/L)	NA	<38 ng/mL	NA
Mar 1, 2014	75 mcg/88 mcg Alternating daily	80.09 µIU/mL (80.09 mIU/L)	2.6 pg/mL (3.3 pmol/L)	1.1 ng/dL (14.2 pmol/L)	NA	9.9 ng/mL (9.9 µg/L)	<20 IU/mL (<20 kIU/L)	NA	71%
Apr 12, 2018	88 mcg daily	68.5 µIU/mL (68.5 mIU/L)	2.3 pg/mL (3.0 pmol/L)	0.97 ng/dL (12.5 pmol/L)	<2.0 ng/mL (<2.0 µg/L)	4.5 ng/mL (4.5 µg/L)	1.3 IU/mL (1.3 kIU/L)	NA	NA
Apr 13, 2022	75 mcg daily	45.7 µIU/mL (45.7 mIU/L)	NA	1.05 ng/dL (13.5 pmol/L)	NA	2.8 ng/mL (2.8 µg/L)	<1.0 IU/mL (<1.0 kIU/L)	NA	NA
Oct 19, 2022	75 mcg daily	85.1 µIU/mL (85.1 mIU/L)	NA	0.96 ng/dL (12.4 pmol/L)	NA	4.6 ng/mL (4.6 µg/L)	<1.0 IU/mL (<1.0 kIU/L)	NA	NA
Oct 16, 2024	75 mcg daily	59.43 µIU/mL (59.42 mIU/L)	NA	0.80 (10.3 pmol/L)	NA	NA	NA	NA	NA

**Abbreviations**: Ab, antibody; IMA, immunometric assay; NA, not available; RIA, radioimmunoassay; T3, triiodothyronine; T4, thyroxine; TSH, thyroid stimulating hormone

## Treatment

In 1977 the patient was started on phenelzine 15 mg 3 times a day, with notable improvement in hyperactivity, heart palpitations, tachycardia, and anxiety. Two years later, he began thyroid replacement therapy for iatrogenic hypothyroidism with levothyroxine at 75 mcg daily. In August 2018, the levothyroxine dose was adjusted to 75 mcg alternating with 88 mcg daily with a maximal dose of 100 mcg alternating with 88 mcg daily achieved by December. His current dose is 75 mcg daily with dosage alteration driven by symptomatology, not TSH values. The patient remains on phenelzine 15 mg 3 times a day, as its discontinuation is associated with a return of heart palpitations, hyperactivity, and restlessness.

## Outcome and follow-up

The patient is monitored by endocrinology with bi-annual evaluations of TSH and FT4 levels. The patient has yet to undergo genetic sequencing to confirm a specific thyroid hormone receptor beta (*THRB*) pathogenic variant due to cost prohibition and low likelihood of altering current medical management. The patient remains asymptomatic with steady T4 despite varying levels of TSH.

## Discussion

We report the clinical and biochemical features of a patient with suspected PRTH who was initially misdiagnosed with hyperthyroidism and subsequently underwent multiple unsuccessful medical and surgical treatments.

RTH is characterized by insensitivity to TH at target tissues. RTH is genetically linked to pathogenic variations in the *THRB* gene [5-9]. There are 2 main isoforms within the *THRB* gene produced via alternative splicing: *TRβ1* and *TRβ2* causing GRTH and PRTH, respectively [3, 6, 8-10]. Pathogenic variations in *TRβ1* are localized to the brain, liver, and kidney while pathogenic variations in *TRβ2* are localized to the hypothalamus, pituitary gland, retina, and cochlea [7]. Pathogenic variations in *THRB* typically demonstrate autosomal dominant inheritance; however, a subpopulation of patients with RTH have autosomal recessive or somatic variations [7-10]. Since its first description, there have been more than 160 different pathogenic variants linked to RTH [4, 7].

The clinical presentation varies based on the specific variations within the TH receptor [8]. Newborns with homozygous pathogenic variations in *THRB* are often identified by neonatal screening with abnormal levels of FT4, TSH, or both [3]. However, RTH has gone undetected at childbirth in patients with heterozygous pathogenic variations due to minimal alterations in levels of FT4 and TSH with no prominent clinical symptoms [3]. Patients with untreated RTH often present later in life with variable symptomatology based on their TH levels and degree of resistance.

RTH is divided into 2 clinical phenotypes, PRTH and GRTH, according to the location of TH resistance. GRTH is characterized by resistance to TH in the pituitary and peripheral tissues; however, compensatory increases in both TH and TSH typically result in an asymptomatic presentation [8]. Patients with hypothyroidism present with goiter, fatigue, somnolence, depression, and bradycardia, while patients with hyperthyroidism present with tachycardia, hyperactivity, short stature, and altered bone age [11]. Hypothyroidism can also occur if euthyroid patients with GRTH are treated with anti-thyroid medications, as their bodies have already developed compensatory mechanisms for overcoming thyroid hormone resistance via elevations in FT4 and TSH [2].

Individuals with PRTH have selective TH resistance in the pituitary gland. This results in decreased feedback on the pituitary gland and subsequent overproduction of TH [2]. The elevated TH manifests with symptoms of overt hyperthyroidism with supranormal or elevated TSH. For those with suspected thyroid dysfunction and unclear laboratory testing, the presence of Woltman sign, or the delay in the relaxation phase of the Achilles reflex, may prove beneficial.

RTH should be suspected in patients who present with elevated TH, particularly FT4, and an inappropriately nonsuppressed TSH. Patients with discordant thyroid levels should undergo workup for coexisting autoimmune thyroid disorders given their increased prevalence in RTH patients [12-14]. The 2 most prevalent mimickers of RTH, assay interference and TSH-secreting adenoma (TSHoma), should be excluded. Assay interference presents with similar discordant thyroid levels secondary to autoantibodies, excess biotin, or Macro-TSH molecules [15]. Assay interference is excluded by autoantibody screenings, sample dilution, or different immunoassays [15].

The other principal condition to consider is a TSHoma. Initial testing to differentiate a TSHoma from RTH includes a thyrotropin-releasing hormone stimulation test where TSHomas do not exhibit TSH excitability, and a L-triiodothyronine inhibition test where supra-physiologic levels of T3 fail to suppress TSH production [16]. For inconclusive results, a somatostatin inhibition test with a significant decrease in TSH levels can support a TSHoma diagnosis [17]. Pituitary MRI may identify larger TSHomas, although reliance on imaging alone is unreliable as case reports of concurrent RTH and TSHoma have been documented [14, 16]. Once these conditions are excluded, genetic sequencing of the *THRB* gene should be completed to identify pathogenic variants in the thyroid hormone receptor.

Once diagnosed, the treatment of PRTH is focused on improving clinical symptoms rather than normalizing FT4 or TSH levels. For many patients, treatment is not necessary as elevated TH compensates for tissue resistance [8]. For patients who exhibit hyperthyroidism, treatment includes beta-blockers or anxiolytics. Triiodothyroacetic acid (TRIAC) is a non-FDA approved T3 metabolite which in some RTH cases has been shown to suppress TSH levels and potentially reduce the incidence of thyroid neoplasia [4, 14]. TRIAC may also decrease thyrotoxic symptoms, although research is ongoing [14]. Anti-thyroid medication should be used selectively in patients with PRTH and concurrent Graves disease [8]. Thyroidectomy and radioactive iodine therapy are contraindicated and can result in lifelong TH supplementation [3].

**Figure 1:**
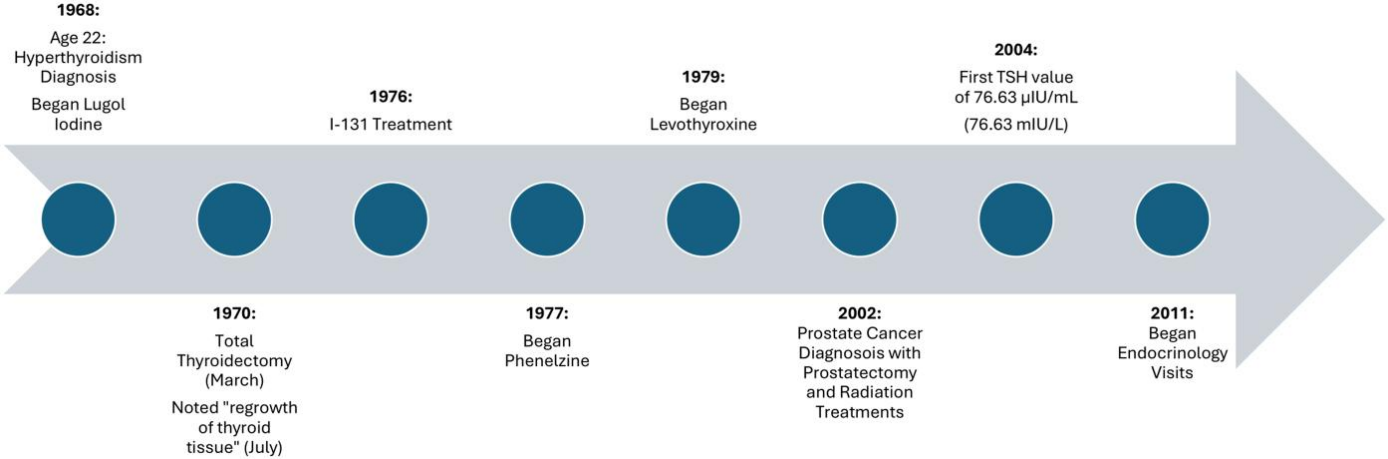
Timeline of major clinical events. Abbreviations: I-131, iodine-131; TSH, thyroid stimulating hormone.

The patient's treatment pathway is summarized in Fig. 1. Of note, the thyroid tissue of this patient regrew within 4 months of total thyroidectomy. While total thyroidectomy is typically a definitive cure for hyperthyroidism, literature suggests functional goiter recurrence can occur in rare cases [18]. In one study of patients with functional thyroid regrowth following thyroidectomy, residual thyroid tissue regrew within the embryologic thyroglossal duct and tubercle of Zuckerkandl in 50%, and 80% to 90%, of patients, respectively [19]. The physiology behind this regeneration remains poorly understood. In animal models, thyroidectomy was shown to alter gene expression within residual thyroid cells, potentially triggering regeneration [20]. We hypothesize that progenitor thyroid cells left behind during surgery rapidly regenerated secondary to prolonged growth stimulation by elevated TSH levels, recreating functional thyroid tissue.

An additional unique aspect of this patient's treatment course is the continued reliance on phenelzine for relief of hyperthyroid symptoms. Historically, radioactive iodine ablation has an overall cure rate of around 87% for hyperthyroidism [21]; however, it did little to suppress this patient's hyperactivity and palpitations. Rather, medical management with phenelzine was effective at decreasing these symptoms. Beyond the anxiolytic properties of phenelzine, literature is sparse regarding the use of monoamine oxidase inhibitors in hyperthyroidism. One small scale study analyzed the effect of short-term phenelzine on TH levels in depressed patients and showed no significant reduction in peripheral TH levels [22]. Attempts at tapering phenelzine have resulted in recurrence of hyperthyroid symptomatology, despite no clear pharmacologic mechanism.

## Learning points

- RTH should be included in the differential for patients with persistently elevated TSH or atypical FT3 and FT4 levels. Common mimickers should be excluded before performing invasive treatments.
- Patients diagnosed with RTH that inadvertently underwent thyroidectomy and/or radioactive iodine treatment benefit from titration of thyroid replacement therapy based on symptomatology.
- Functional thyroid regrowth remains a rare phenomenon, likely secondary to residual embryological tissue, incomplete surgical excision, alterations in residual thyroid gene expression, and perhaps elevated TSH stimulation.

## Data Availability

Original data generated and analyzed during this study are included in this published article.
